# Effect of Chitosan Coating with Cinnamon Oil on the Quality and Physiological Attributes of China Jujube Fruits

**DOI:** 10.1155/2015/835151

**Published:** 2015-09-30

**Authors:** Yage Xing, Hongbin Lin, Dong Cao, Qinglian Xu, Wenfeng Han, Ranran Wang, Zhenming Che, Xihong Li

**Affiliations:** ^1^Key Laboratory of Grain and Oil Processing and Food Safety under the Supervision of Sichuan Province, College of Food and Bioengineering, Xihua University, Chengdu 610039, China; ^2^School of Food Engineering, Luohe College of Vocational Technology, Luohe 462000, China; ^3^School of Food Engineering and Biotechnology, Tianjin University of Science & Technology, Tianjin 300457, China

## Abstract

Effects of chitosan coating with cinnamon oil on the physiological attributes and preservation quality of China jujube fruits during storage at 4°C for 60 days were investigated. Results indicated that weight loss and decay of jujube fruits were significantly reduced by chitosan-oil coating during the period of 60-day storage, which also exhibited a quite beneficial effect on maintaining the sensory quality for jujube fruits. Meanwhile, the contents of vitamin C and titratable acid decreased to 3.08 mg·g^−1^ and 0.342% for the fruits treated by chitosan-oil coating (1.0% + 0.10%), respectively. Polyphenol oxidase, superoxide dismutase, and peroxidase activities were 13.40 U·g^−1^, 14.53 U·g^−1^, and 63.6 U·g^−1^ at the end of storage, respectively. The contents of total soluble phenolics and MDA were 34.51 mg·g^−1^ and 19.43 *μ*mol·g^−1^ for the combined coating treated samples and control fruits, respectively. These results suggested that the chitosan-oil coating might be recognized as one efficiency technology on the preservation quality of jujube fruits during the storage time.

## 1. Introduction

Jujube is a native fruit in China with the long history of over 2500 years [1–3]. Lingwu Long Jujube fruit (*Ziziphus jujuba* Mill cv.), one of the local colored fresh-eat jujube fruits in Ningxia, has the special flavor and could provide various sources of nutrition for costumer. However, the further industrial and economic development of jujube fruit is limited because of its short shelf-life, only 15-day storage at room temperature, and severe losses during storage [3–5]. Moreover, the ripening and accelerated senescence could induce the short storage life and bad quality of jujubes for the long time of storage. Although the utilization of fungicides is the primary means of controlling postharvest diseases and decay, the problems of negative impact for using excessively on the costumer health have prompted awareness recently. The investigation for the safe alternatives by many researchers is increasing on the storage of fruits and vegetables, such as the application of chitosan and essential oils, which has been widely used due to its beneficial effects for delaying senescence and maintaining quality [5].

The possibility of edible coating to carry essential oil is being studied because the oil as antimicrobial agent can be released slowly from coating carriers to the food surface and maintain the option oil concentrations in the microenvironment. Chitosan is a natural carbohydrate biopolymer with different functional groups (primary -OH, secondary -OH, and NH) [6, 7], which is applied widely in the storage of fruits and vegetables because of its film-forming and antimicrobial activities [8]. As the result reported by Romanazzi et al. [9], blue mold rots of sweet cherry were reduced and controlled by chitosan dipping. The investigation of Chien et al. [10] demonstrated that the treatment of chitosan coating on the citrus fruit exhibited the controlling effect on the decay of fruit during the storage time. Furthermore, essential oils incorporated into the chitosan coating as the carrier could greatly enhance its antimicrobial property for application on the storage of fruits [11, 12].

Application of essential oils, such as cinnamon oil and clove oil, has attracted increasing interest due to its better antimicrobial activity against bacteria, yeasts, and moulds and higher safety to environment and costumer [12–16]. Xing et al. [17] reported that the treatment of chitosan-oil coating could provide the better effect on the quality and decay of sweet peppers during storage. Furthermore, the conidial germination and mycelial growth of all fungi on banana could also be inhibited by cinnamon oil [12]. The investigation of Xing et al. [3] also reported that, in the* in vivo* study,* Penicillium citrinum* growth on wound inoculated fruits could be completely controlled by the coating with cinnamon oil at 2.0% [3]. As reported by Xing et al. [16], the combined application of antibrowning agents, oil fumigation, and moderate vacuum packaging (MVP) was reported to delay the microbiological deterioration and prolonged the shelf-life of lotus root slices [16]. However, there is no published investigation regarding the effect of chitosan coating with cinnamon oil on the physiological attributes and preservation quality of jujube fruits without wound inoculation during the storage period.

The objective of this investigation was to evaluate the effects of chitosan-oil coating on physiological attributes and preservation quality of Lingwu Long Jujube fruits. The decay, weight loss, sensory acceptability, titratable acidity, and vitamin C content were determined for jujube fruits with different treatments during storage at 4°C for 60 days, respectively. Furthermore, peroxidase (POD), polyphenol oxidase (PPO) and superoxide dismutase (SOD) activity, the total soluble phenolic content, malondialdehyde (MDA) content, and membrane permeability in fruits were also evaluated during the whole storage time, respectively.

## 2. Materials and Methods

### 2.1. Preparation of Chitosan-Based Coating

The chitosan coating was prepared as the method reported by Xing et al. [3] and Xing et al. [17]. The chitosan (1.0%) solution contained glycerol (0.75%) and acetic acid (0.5%) was stirred at room temperature for 1 h (chitosan, deacetylated ≥95%, Jinan Haidebei Marline Bioengineering Co. Ltd., Jinan, China). Then, cinnamon oil (Xianghui Biotechnology Co. Ltd., Shanghai, China) with the concentration of 0 and 0.10% mixed with Tween 80 (0.2%) was added and stirred for another 30 min, respectively. Food grade dimethyl silicon oil (0.1%) was added as an antifoaming agent. The final solution was homogenized at 21600 rpm under aseptic conditions for 1 min. The solution without the addition of cinnamon oil and chitosan was prepared as the control coating. The obtained coating could be used after standing for 1 h.

The harvested Lingwu Jujube fruits (*Ziziphus jujuba* Mill cv.) were chosen with absence of disease infection or physical injuries firstly. These chosen fruits were divided into four groups (1200 fruits/each group) after being surface-disinfected with sodium hypochlorite (2%, w/v) for 3 min, air-dried, and precooled at 0°C for 12 h. Jujube fruits were dipped into four different treatment coating solutions for 5 min (1, control; 2, chitosan coating (1.0%); 3, oil (0.10%) treatment; 4, chitosan (1.0%) + oil (0.10%)), respectively. Furthermore, the coated fruits were well dispersed in the tray and packaged with a polypropylene film (80 cm × 60 cm, 120 fruits/bag). And then the packaged fruits were stored at 4°C after being kept over a plastic sieve for 5 min and air-dried for 60 min.

### 2.2. Determination of Inhibitory Zone of Chitosan-Oil Coating and Cinnamon Oil

The antimicrobial activity of different coating was evaluated using the agar diffusion method, respectively [4, 15, 18, 19]. For* Escherichia coli* and* Staphylococcus aureus*, the sample disc (*D* = 10 mm) impregnated with 10 *μ*L different solution was placed on LB plate, which had been spread with 100 *μ*L bacterial suspension with 10^4^–10^5^ CFU·mL^−1^ of tested bacteria, respectively. For antifungal activity, the disc (*D* = 10 mm) impregnated with 10 *μ*L different solution was placed on potato dextrose agar plate, which had been spread with 100 *μ*L bacterial suspension with 10^4^–10^5^ conidia·mL^−1^ of* Rhizopus nigricans*,* Penicillium citrinum*,* Aspergillus flavus*, and* Penicillium expansum*, respectively. The plates were incubated at 37°C for 24 h for bacteria and at 28°C for 72 h for fungi in the incubation chamber, respectively, and then the diameters of “inhibition zone” were measured.

### 2.3. Morphological Observation by Atomic Force Microscopy and Scanning Electron Microscopy

The dried coating piece sample was observed by AFM with a 3.51 *μ*m vertical range and an 80 mm × 80 mm scan size (atomic force microscopy) in tapping mode using a multimode JSPM-5200 AFM (JEOL, Japan), which was equipped with the Si3N4 cantilevered scanner [17, 20]. Furthermore, after being placed on the stub using two-sided adhesive tapes and Pt sputtering, the dried coating was also observed by SEM (scanning electron microscopy) at a voltage of 5 kV acceleration [2].

### 2.4. Fruits Decay Rate, Sensory Acceptability, Weight Loss, Titratable Acidity, and Vitamin C Content

The total count and decayed count of fruits were counted during the storage time, respectively. The decay rate was calculated as follows: fruit decay rate (%) = (the decayed count of fruits × 100/the total count of fruits) [3]. On the other hand, after being surfaced and disinfected and air-dried, sensory acceptability of jujube fruits was evaluated and rated on a nine-point hedonic scale (9, excellent; 7, very good; 5, good; 3, fair; and 1, poor) by six panelists in random [3, 21]. The weight loss, titratable acidity, and vitamin C in samples were determined as the method developed by Xing et al. [17], Odriozola-Serrano et al. [22], and Xing et al. [16].

### 2.5. PPO, POD, and SOD Activities

The PPO activity in jujube fruits was determined according to the method conducted by Xing et al. [3] and Xing et al. [17]. Fruits tissue (1.0 g) was homogenized in an ice bath with 2.0 mL extraction buffer (sodium phosphate buffer with 20 g·kg^−1^ polyvinylpolypyrrolidone, 0.2 mol·L^−1^, pH 6.8, 4°C) for 4 min. The obtained solution was centrifuged at 4°C with 13,000 g for 15 min. The reaction solution consisted of 2.9 mL substrate solution (0.02 mol·L^−1^ catechol in 0.05 mol·L^−1^ phosphate buffer, pH 6.5) and 0.1 mL crude extract. The catechol oxidation rate was evaluated at 420 nm for 2 min at room temperature. And the activity unit was defined as an increase of 0.0001 in absorbance for one minute.

POD and SOD activities were evaluated as the method reported by Xu et al. (2009) and Xing et al. (2011a) [17, 23]. 2.5 g Fruit tissue was homogenized in 10 mL PBS (25 mmol·L^−1^, pH 7.8, containing 1 mmol·L^−1^ EDTA and 0.8 g·L^−1^ PVPP) and then centrifuged at 4°C with 13,000 g for 20 min. For the determination of POD activity in fruits, 0.5 mL enzyme extract was incubated at 30°C in 2 mL buffered substrate (pH 6.4, 100 mmol·L^−1^ sodium phosphate and 8 mmol·L^−1^ guaiacol) for 5 min and the absorbance measured at 465 nm every 30 s for 120 s after adding 1 mL of H_2_O_2_ with the concentration of 24 mmol·L^−1^. POD activity in jujube fruits was expressed as U·g^−1^ (U = 0.01 D-absorbance_465 nm_ min^−1^). For SOD determination, the reaction mixture (3 mL) consisted of 13 mmol·L^−1^ methionine, 50 mmol·L^−1^ sodium phosphate buffer (pH 7.8), 10 *μ*mol·L^−1^ EDTA, 75 *μ*mol·L^−1^ nitroblue tetrazolium, 2 *μ*mol·L^−1^ riboflavin, and 0.1 mL enzyme extract. The mixtures solutions were illuminated for 10 min by light (60 *μ*mol·m^−2^·s^−1^). Identical solutions held in the dark served as blanks. The absorbance was measured at 560 nm and SOD activity was expressed as U·g^−1^. One enzyme unit was recognized as the enzyme volume corresponding to 50% inhibition of nitroblue tetrazolium reduction at 560 nm.

### 2.6. Total Soluble Phenolics Content, MDA Content, and Membrane Permeability Determination

Total soluble phenolics content in jujube fruit was determined according to the method of Xing et al. [3]. 1 g fruit tissue was homogenized and centrifuged at 12,000 g for 15 min after being frozen in 2.5 mL of 95% ethanol for 72 h. 1 mL obtained supernatant was mixed with 5 mL distilled water, 1 mL 95% ethanol, and 0.5 mL of 50% (1 N) Folin-Ciocalteu phenol reagent. Furthermore, 1 mL of 5% (w/v) sodium carbonate was added and followed by brief vortexing to mix after being incubated at room temperature for 5 min. And then, the absorbance at 725 nm of the reaction mixture was determined and converted to mg·g^−1^ (phenolics) tissue after being vortexed and incubated for 1 h in the dark. In this investigation, total phenolics content was standardized against gallic acid. On the other hand, TBARS expressed as MDA equivalents and membrane permeability as relative leakage rate were determined according to the method of Xing et al. [17]. The further details of determined method were not described here.

### 2.7. Statistical Analysis

The obtained values were calculated as mean ± S.D. (*n* = 3). The data for antimicrobial activity and fruits decay rate were analyzed by SPSS 13.0 software with one-way analysis of variance and Student-Newman-Keuls test. The differences were recognized as significant (*P* < 0.05).

## 3. Results and Discussion

### 3.1. Antimicrobial Activity of Chitosan-Oil Coating

**Figure 1:**
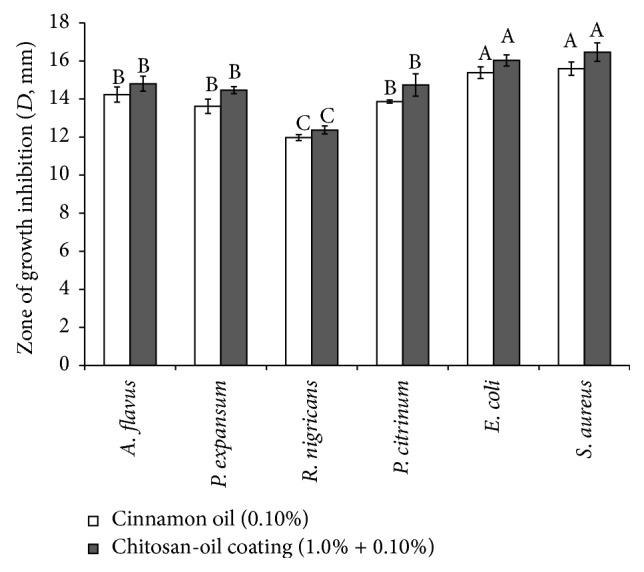
Antimicrobial activity of cinnamon oil and chitosan-oil coating.

Antimicrobial properties of different coating against fungi and bacteria were investigated. As shown in Figure 1, the zone diameters of inhibition of chitosan-oil coating and cinnamon oil coating against* A. flavus*,* P. expansum*,* R. nigricans*, and* P. citrinum* were 14.23 mm, 13.60 mm, 11.97 mm, 13.48 mm and 14.80 mm, 14.47 mm, 12.37 mm, and 16.46 mm, respectively. Furthermore, the diameters of inhibition zone of chitosan-oil coating against* E. coli* and* S. aureus* were 15.39 mm and 15.59 mm, respectively, which were 16.03 mm and 16.46 mm for cinnamon oil coating. Result indicated that the activity of different coating against bacteria might be higher than that against fungi in this investigation. The lowest and highest activities of chitosan-oil coating and cinnamon oil coating were observed against* R. nigricans* and* S. aureus*, respectively.

Better quality without decay is one of the key factors for evaluating the effect of fruits storage [16, 24]. The application of chitosan coating as the carrier could be due to its good antimicrobial activity and film-forming property with micropores [3, 25]. The investigation of Xing et al. [3] demonstrated that the chitosan-oil coating exhibited the best control effect against the growth of* P. citrinum* on wound inoculated fruits. Result also suggested that the antimicrobial properties of chitosan coating were significantly improved by the addition of cinnamon oil [3, 15, 23]. The result reported by Tzortzakis [14] also indicated that the controlled effects of cinnamon oil on the spore germination in* Rhizopus stolonifer* were related to the oil concentration. Xing et al. [15] also showed that cinnamon oil exhibited the good antifungal activity against* A. flavus*,* P. expansum,* and* R. nigricans*. Furthermore, the investigation of Xing et al. [15] indicated that cinnamon oil (2.0%) showed complete control on the growth of fungi inoculated in wound jujube fruits. These results revealed that the chitosan-oil coating has a good potential to be a natural antimicrobial agent on the applications of fruit storage.

### 3.2. Weight Loss, Fruit Decay, and Sensory Acceptability

**Figure 2:**
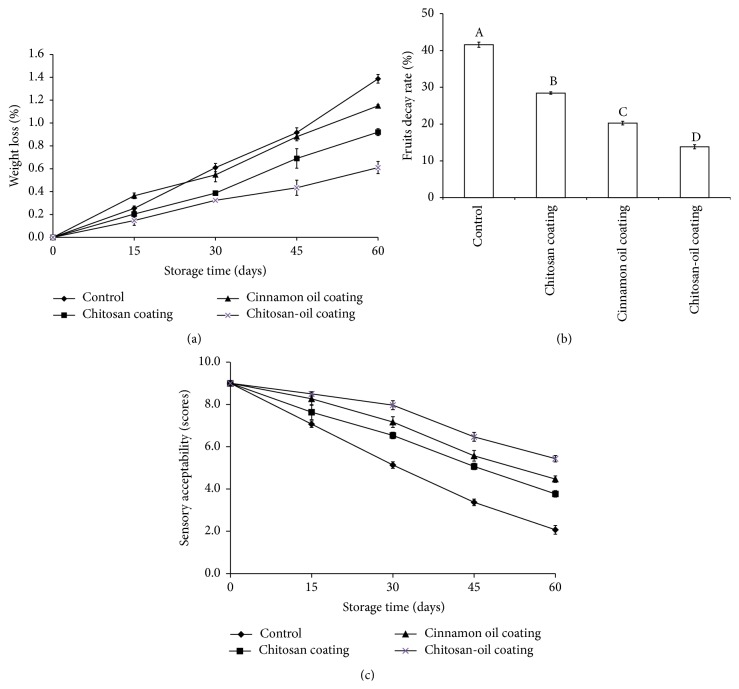
Weight loss (a), disease incidence (b), and sensory acceptability (c) of the samples during 60-day storage at 4°C.

Water loss as the main part of weight loss is one of the quite important reasons that could affect the quality of fresh fruit products [24]. According to the result in Figure 2(a), jujube fruits treated by the combined coating presented the lowest weight loss (0.53%) after 60 days of storage at 4°C. However, the control fruits presented the highest weight loss (1.39%) after storage. Moreover, the effect of different coating on the postharvest decay of jujube fruits is shown in Figure 2(b). The control jujube fruits started decaying before day 15 (data was not shown) and reached 41.6% for the decay rate of fruits at the end of storage. Meanwhile, the jujube with chitosan-oil coating showed little fruit decay during the first 15 days of storage. The fruit decay rate of fruits treated by chitosan-oil coating was only 13.83%, which was significantly lower than that of the control on day 60. On the other hand, result shown in Figure 2(c) indicated that the chitosan coatings enriched with cinnamon oil at 0.10% could not produce undesirable sensory properties for fruits during the whole storage. The samples treated by chitosan-oil coating exhibited the highest sensory acceptability (5.43) among all the treatments. But the sensory acceptability of the control samples was only 2.07.

**Figure 3:**
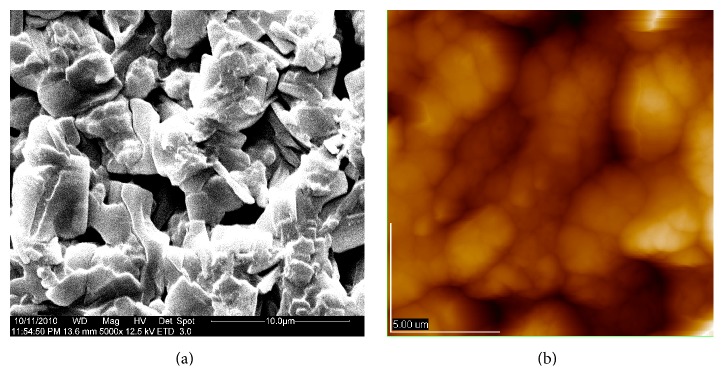
Microstructure of chitosan coating observed by SEM and AFM.

Result indicated that the chitosan-oil coating could provide the better control effect on the weight loss and fruit decay and provide better sensory acceptability for fruits during storage. The fruit samples treated by the combined coating presented lower weight loss than that of the control fruits. Chitosan coating could serve as a protective layer for water loss during the storage of postharvest fruits [3, 17, 26]. The microstructure of chitosan coating was observed by SEM and AFM. This is because micropores as the microperforated panels for chitosan coating used to wrap jujube fruits, as shown in Figure 3, could moderate probably letting O_2_ and CO_2_ to pass through and prevent the quick loss of water molecules from the surface of fruits. These results were consistent with the observation of Xing et al. [3]; the minimum weight loss was found to occur in the fruits treated with the combined coating with chitosan and cinnamon oil. The combined coating could help in slowing the ripening rate and reducing the weight loss of fruits [27, 28]. Liu et al. [29] reported that the control effect of chitosan on blue mold was also observed in tomato fruit [29]. Our study also showed that the controlling activity of chitosan coating on disease decay was enhanced by the addition of cinnamon oil [3, 12, 14, 15]. This combined beneficial function for controlling decay of jujube fruits could be due to the antimicrobial activity of chitosan-oil coating and the control effect of chitosan coating as a barrier on the loss of cinnamon oil [10, 30, 31]. On the other hand, the appearance of fruits is a primary factor in quality judgment for the consumers concerned. As reported by Ojagh et al. [13], the chitosan-based coating incorporated with cinnamon oil was found to keep the good quality on the fish samples during storage. The investigation of Xing et al. [15] reported that the samples treated by chitosan coating and MAP exhibited the highest overall visual quality scores of fresh-cut lotus root at the end of storage [15, 32]. Furthermore, Xing et al. [17] also reported that the chitosan-oil coating could keep the sensory quality of sweet peppers. In conclusion, chitosan coating with cinnamon oil at appropriate dose could be considered as a safe alternative for keeping fruits quality.

### 3.3. Titratable Acidity and Vitamin C Content

**Figure 4:**
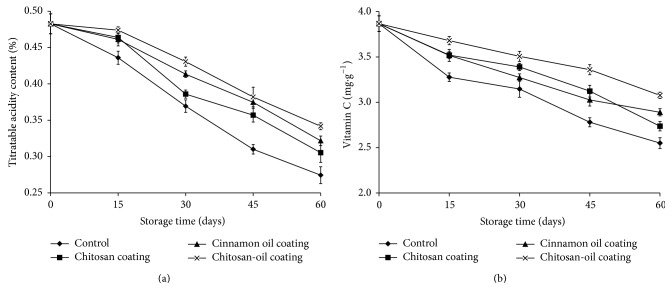
Titratable acidity contents (a) and vitamin C contents (b) in the samples during 60-day storage at 4°C.

The effects of different coating on the titratable acidity (TA) in jujube fruits were shown in Figure 4(a). Results revealed that titratable acidity in fruit samples was found to decrease along the storage time. TA content in the control samples was significantly lower than the initial value at the end of storage at 4°C. After 60 days of storage, the untreated jujube fruits presented the highest decrease in TA, while the fruits treated with the combined coating showed the lowest decrease. TA content in fruits treated by the chitosan-oil coating was only 0.34% at day 60. Furthermore, the effect of chitosan-oil coating on the vitamin C content was also evaluated during the storage time. The results in Figure 4(b) illustrated that a significant decrease in vitamin C values of chitosan-oil coated fruits was observed along with the storage period. After storage at 4°C for 60 days, vitamin C values of the fruits tread with the chitosan-oil coating and the control samples were 3.08 mg·g^−1^ and 2.55 mg·g^−1^, respectively. The decrease rate in vitamin C was significantly lower in coated fruits compared to that in the control samples.

These results illustrated that the contents of TA and vitamin C in jujube fruits with chitosan-oil coating were the highest among all treatments during the storage time. The high TA content could be attributed to the chitosan coating, which could control the permeability of CO_2_ and O_2_ and further slow the ripening rate of fruits. The relation between TA and ripening rate could be further investigated in the next word [16, 24]. Chitosan-based oil coating could reduce the ripening rate of fruits and the substrate for respiration responses such as organic acids [16, 24, 26]. Xing et al. [16] reduced respiration rate of fruits that may be reflected in lower changes in titratable acidity [33, 34]. Furthermore, the reason for high vitamin C content in coated fruits during the storage time could be attributed to the combined function of chitosan and cinnamon oil coating, which could significantly slow the ripening rate and provide the preserve for vitamin C in jujube fruits [3, 17]. According to the investigation of Xing et al. [16], the loss of vitamin C can be greatly favored by the presence of O_2_. This is because chitosan coating could reduce O_2_ content in the microenvironment and cinnamon oil as the antibrowning agents provide better preserve on the vitamin C contents in fruits. These similar results have been reported for other cultivars such as carrot and fresh vegetable juice [35]. Also, vitamin C in the jujube fruits might be protected by antioxidant activity of cinnamon oil [36–38]. As reported by Xing et al. [17], at the end of storage, the highest vitamin C content was observed in peppers treated by chitosan-oil coating. Cinnamon oil in the chitosan coating as the carrier could inhibit the vitamin C loss by acting as an abiotic elicitor [3, 15, 17, 39–41].

### 3.4. PPO, POD, and SOD Activity in Jujube Fruits

**Figure 5:**
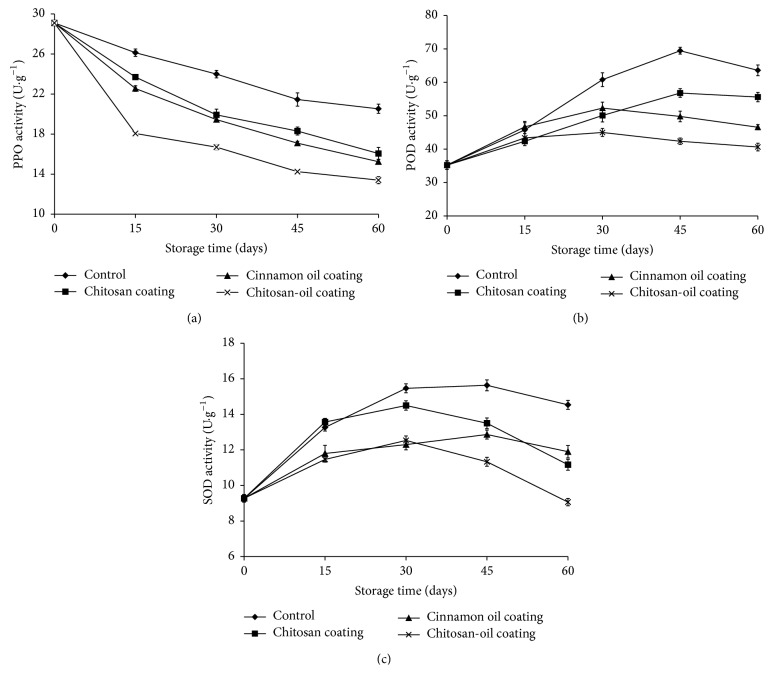
PPO (a), POD (b), and COD (c) activities in the samples during 60-day storage at 4°C.

PPO, POD, and SOD activities in jujube fruits treated by different coating during storage at 4°C for 60 days were investigated. Result presented in Figure 5(a) illustrated that, at zero time, high PPO activity was observed (29.10 U·g^−1^). While after 60 days of storage at 4°C, the activity of the control samples was 20.53 U·g^−1^, which decreased to 13.40 U·g^−1^ in the jujube fruits treated with the chitosan-oil coating. Furthermore, as revealed in Figure 5(b), POD activity increased in both control and chitosan-oil coating treated fruits during the storage time. The higher POD activity in chitosan-oil treated fruit at the end of storage indicated that chitosan coating can effectively inhibit the POD activity during the storage time. At zero time, high POD activity was observed (35.23 U·g^−1^). While after 60 days of storage at 4°C, the activities were 40.63 U·g^−1^ and 63.60 U·g^−1^ in control samples and the jujube fruits treated with the chitosan-oil coating, respectively. Senescence is considered to be associated with the defensive system, such as SOD enzymes, in the fruits during storage. As can be seen in Figure 5(c), SOD activities in the all treated groups showed an increased trend first and decreased thereafter. However, SOD activity was higher than that in the coating treated fruits after 60 days, which indicates that chitosan coating can effectively inhibit the SOD activity during the storage time. As shown in Figure 5(c), the SOD activity reached 9.27 U·g^−1^ in jujube fruits before being treated. However, the activity was 14.53 U·g^−1^ in the jujube fruits treated with the chitosan-oil coating, which was only 9.07 U·g^−1^ in the control fruit samples at the end of storage. Chitosan (1.0%) coating with cinnamon oil (0.10%) was the most active one in enhancing the SOD activity in fruit.

The results revealed that the effect of chitosan-oil treatments on the PPO activity in the jujube fruit was found. This phenomenon was consistent with the observation reported by Badawy and Rabea [8], who reported that chitosan coating could provide the ability to remove the metal ions and has the potential effect used to inhibit the PPO activity in litchi fruit [8, 42, 43]. The coordinated action of SOD and POD, two important oxyradical detoxification enzymes in fruits, could help to reduce the oxidative damage in regeneration of ascorbate and glutathione metabolites [44–46]. The increased activities of SOD and POD in jujube fruits may be induced by the complex coating of chitosan and cinnamon oil, which could possibly be of benefit to induce the disease resistance in fruits during the storage time [17, 30, 44]. Chitosan coating could induce the activities of defense-related enzymes and promote the protection for fruits [30]. Moreover, the lower PPO activity may be also due to the antioxidant activity of cinnamon oil in the chitosan coating carrier [14, 38, 43, 47]. In the investigation conducted by Xing et al. [32], the inhibitory effect on PPO activity in the treated samples should be due to the cinnamon oil incorporated into chitosan-based coating [37].

### 3.5. Total Soluble Phenolics Content

**Figure 6:**
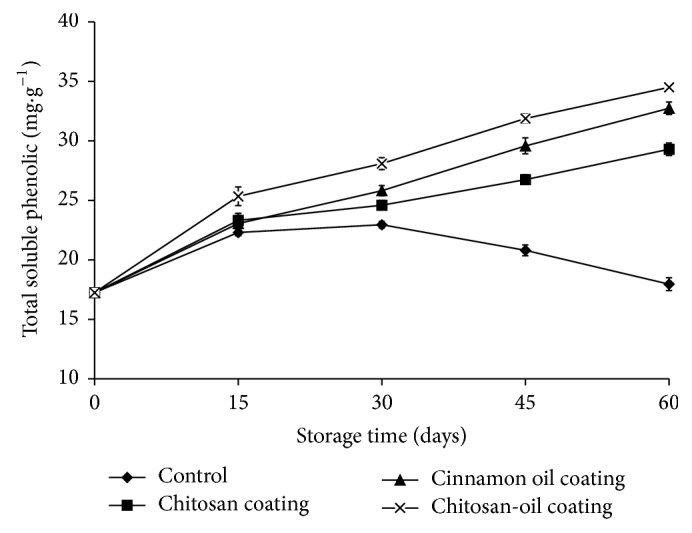
Total soluble phenolic contents in the samples during 60-day storage at 4°C.

Changes of total soluble phenolics content in jujube fruits treated with different coating are shown in Figure 6. The total phenolics in all treatments accumulated gradually except for the control samples during the storage time at 4°C for the first 30 days. However, it was decreased for total phenolics content in fruits after 30 days of storage. Total phenolics content in jujube fruit was up to 34.51 mg·g^−1^ of tissue in the samples treated with chitosan-oil coating, which was only 17.95 mg·g^−1^ of tissue for the control fruits. The complex coating of chitosan and cinnamon oil might be the most active one in increasing the total soluble phenolic content of jujube fruits during the storage period.

The first increased trend of total soluble phenolic compounds in chitosan-treated jujube fruits might be because chitosan has an effect on the activation of plant defense responses [8]. The increase in phenolic substances following chitosan application has been observed by other researchers. Xing et al. [16] reported that, during the storage of fruits, the content of some phenolic compounds as the substrates of PPO was found to decrease because of the oxidation by PPO [3]. This similar result was in agreement with the observations of Liu et al. [29]. These results also suggested that the increase of phenolic compounds and antioxidant enzyme activities in fruits induced by chitosan-oil coating might be of benefit to improve the disease resistance for jujube fruits [44]. On the other hand, the first increased trend of total soluble phenolic compounds might be also due to the other component in chitosan coating, cinnamon oil, which could be able to delay, retard, or prevent oxidation processes of phenolic compounds by reacting with free radicals, chelating metals, and acting as oxygen scavengers [37].

### 3.6. MDA Content and Relative Conductivity

**Figure 7:**
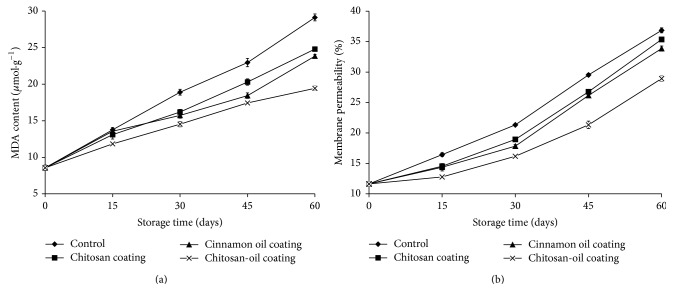
MDA content (a) and relative conductivity (b) in the samples during 60-day storage at 4°C.

Malondialdehyde (MDA) as the final product of lipid peroxidation is always used as one index for oxidative damage of fruit tissue cell [44]. The continuous increase in MDA content was observed both in control and in the coating treated fruits during storage at 4°C (Figure 7(a)). However, at the end of storage, the MDA content in control samples and the chitosan-oil coated fruits were up to 29.13 *μ*mol·g^−1^ and 19.43 *μ*mol·g^−1^, respectively. During the storage time, MDA content in the jujube fruits treated with chitosan-oil coating increased slower than in the pulp of the control and the chitosan coating treated fruits. Similar results were obtained from the electrolyte leakage contents of jujube fruit samples, as shown in Figure 7(b). There was a continuous increase in MDA content, both in control and coating-treated fruits during storage period (Figure 7(b)). The relative conductivity reached 36.87% and 28.93% in the control samples and fruits treated with chitosan-oil coating at the end of storage, respectively.

This result may be due to the chitosan coating, which has a potential for inducing defense system. This is also because of the antioxidant activity of cinnamon extracted oil. Similar results were obtained by Xu et al. [44]; MDA content in the fruit treated with grapefruit seed extract was found to increase slower than that in the control fruit. Membrane permeability, as an indicator of membrane integrity, was also investigated in order to have more information on the stability of cell membrane in fruit tissue [44]. Chitosan-oil coating treated fruits had significantly lower relative leakage rates than the control, indicating that the higher membrane integrity was maintained for fruits during the storage time. This result was consistent with the observation by Xing et al. [17]; the application of chitosan-oil coating could significantly delay the increase of MDA and electrolyte leakage in sweet peppers during the storage time. Results reported by Xing et al. [32] also illustrated that the effect of the treatment combining chitosan-based coating with modified atmosphere packaging was observed in the controlling of MDA accumulation and reducing the oxidative damage of fresh-cut lotus root during storage [48].

## 4. Conclusion

This investigation revealed that the complex coating of chitosan and cinnamon oil could directly control the disease decay and maintain the good sensory acceptability of jujube fruits. Moreover, during the storage time, the treatment of chitosan-oil coating might be to maintain the quality attributes and induce the defense reaction system of jujube fruits. The chitosan-based coating with cinnamon oil could be recognized as the better treatment with regard to inducing several host defense mechanisms. These results in this investigation suggested that chitosan-oil coating (1.0% + 0.10%) kept great quality maintenance and extended the shelf-life of jujube fruit storage at 4°C for 60 days.
